# Head and neck irradiation modulates pharmacokinetics of 5-fluorouracil and cisplatin

**DOI:** 10.1186/1479-5876-11-231

**Published:** 2013-09-26

**Authors:** Chen-Hsi Hsieh, Mei-Ling Hou, Meng-Hsuan Chiang, Hung-Chi Tai, Hui-Ju Tien, Li-Ying Wang, Tung-Hu Tsai, Yu-Jen Chen

**Affiliations:** 1 Division of Radiation Oncology, Department of Radiology, Far Eastern Memorial Hospital, Taipei, Taiwan; 2 Institute of Traditional Medicine, School of Medicine, National Yang-Ming University, 155, Li-Nong Street Section 2, Taipei 112, Taiwan; 3 Department of Medicine, School of Medicine, National Yang-Ming University, Taipei, Taiwan; 4 Department of Radiation Oncology, Mackay Memorial Hospital, 92, Chung-Shan North Road, Taipei, Taiwan; 5 Department of Medical Research, Mackay Memorial Hospital, Taipei, Taiwan; 6 Department of Education and Research, Taipei City Hospital, Taipei, Taiwan; 7 School and Graduate Institute of Physical Therapy, College of Medicine, National Taiwan University, Taipei, Taiwan

**Keywords:** 5-Fluorouracil (5-FU), Cisplatin (CDDP), Concurrent chemoradiation therapy (CCRT), Head and neck, Pharmacokinetics, Radiotherapy

## Abstract

**Background:**

5-fluorouracil (5-FU) and cisplatin (CDDP) are used to enhance radiotherapy (RT) effect for head and neck (HN) cancers. However, the effect of local RT on systemic chemotherapeutics remains unclear. Here, we evaluated the influence of HN irradiation on the pharmacokinetics (PK) of 5-FU and CDDP in rats as experimental model.

**Methods:**

The radiation dose distributions of HN cancer patients were determined for the low dose areas, which are generously deposited around the target volume. Two Gy and 0.5 Gy RT were selected. Single-fraction radiation was delivered to the HN of Sprague–Dawley rats. 5-FU at 100 mg/kg or CDDP at 5 mg/kg was intravenously infused 24 hours after radiation.

**Results:**

Radiation at 2 Gy reduced the area under the plasma concentration *vs.* time curve (AUC) of 5-FU and CDDP by 16% and 29% compared to non-irradiated controls, respectively. This was accompanied by incremental total plasma clearance values. Intriguingly, low dose radiation at 0.5 Gy resulted in a similar pharmacokinetic profile, with a 17% and 33% reduction in the AUC of 5-FU and CDDP, respectively. The changes in AUC of bile, which increases with RT, were opposite to AUC of plasma for both drugs.

**Conclusions:**

The local HN RT could modulate systemic PK of 5-FU and CDDP in rats. This unexpected RT-PK phenomena may provide a reference for adjustment of drug administration and is worthy of further investigation.

**Trial registration:**

ClinicalTrials.gov ID NCT01755585 and NCT01609114

## Background

The concurrent use of chemotherapy during radiation therapy (CCRT) is now the important treatment stratagem as definitive treatment [1] or adjuvant setting for locally advanced head and neck cancer [2,3]. For these cases, 5-Fluorouracil (5-FU) and cisplatin (CDDP) are the most commonly used agents in CCRT to improve the treatment outcome [4-6]. As radiation techniques improving, that three-dimensional conformal radiotherapy (3DCRT), intensity-modulated radiotherapy (IMRT), and image-guided arc therapy such as helical tomotherapy (HT) are applied for cancer treatment worldwide. These are supposed to produce greater target dose conformity and better critical organ sparing effects with lower toxicity to normal tissues [7-11]. However, these dose-painting techniques usually produce a generous low-dose distribution to the torso which biological effect remains unclear [12,13].

Radiation therapy (RT) is classically considered as a local treatment. However, irradiation not only can cause direct DNA damage effects but also can send signals to neighboring cells named as the bystander effects [14,15] or caused longer-range effects called abscopal effects [16]. Recently, we report that abdominal irradiation no matter 0.5 Gy considering as off-target area in clinical practice or 2 Gy, the daily treatment dose, could significantly modulate the systemic pharmacokinetics (PK) of 5-FU in an experimental rat model [17]. Furthermore, our next study confirm that local pelvic irradiation with the liver and kidneys excluded also modulated the systemic PK of 5-FU through stimulating the release of matrix metalloproteinase-8 (MMP-8) [18]. The field of RT in the study is excluded the live and the kidneys [18]. However, there are still a few parts of intestines being involved in the whole pelvic field. Leukocyte infiltration of rat small intestine could be induced by 5-FU [19] and the passive transport and carrier-mediated transport in intestinal also influences uptake of 5-FU [20]. Given that head and neck cancers are common in cancer population and CCRT is widely used in clinical practice, whether the RT-PK phenomena we observed in abdomen and pelvis RT with 5-FU exists in head and neck region is an important issue to be addressed.

CDDP (cis-diammine-dichloro-platinum) is an inorganic molecule, with a central platinum atom surrounded by two chlorine atoms and two ammonia molecules [21]. It is often used as an attractive chemotherapy drug and broadly used for the treatment of various forms of malignant tumors. There are five major characteristics that are considered to be responsible for the cytotoxic effects of cisplatin. As a radiosensitizer, its affinity with the thermalized electron created by radiation induced ionization within the DNA molecule and may lead to irreparable damage to the DNA [22]. Moreover, the inhibition of sublethal damage repair (SLDR) by cisplatin is demonstrated by experiments on oxic mammalian cells [23]. Cisplatin also has the ability to arrest cells in the G2 phase of the cell cycle and, possibly, to induce their death [24-26]. Research showed that a cell’s relative radiosensitivity is determined by the cell cycle phase. Cells are most radiosensitive in the G2/M phase, less sensitive in the G1 phase and least sensitive during the latter part of the S phase [27]. In addition, the suppression of tumour neovascularization by cisplatin has been identified recently and is under further investigation [28].

In the present study, we investigated the effect of head and neck RT, including therapeutic fraction size and off-target dose, on pharmacokinetics of 5-FU and CDDP in rats. The issues of advance in dose-painting techniques and adoption of CCRT did not exist in the past era of conventional 2DRT. Therefore, the conceptual correlation to clinical practice in humans is drawn from point of view of the radiation oncologist in this translational research.

## Methods

### Subjects

Prior to pharmacokinetic analysis in rats, we demonstrated the radiation dose distributions in a head and neck cancer patient to show the dose-painting concept that low dose area generously deposited around high-dose target volume.

### Targeting and treatment planning

Although only treated by one mode of RT, four sets of radiation planning were performed for each patient including that for conventional radiotherapy (2DRT), 3DCRT, IMRT, and HT. The PINNACLE^3^ version 7.6c planning system for the former three modes and the Hi Art Planning system for tomotherapy (Tomotherapy, Inc., Madison, Wisconsin, USA) were used. The treatment fields for 2DRT, 3DCRT, and IMRT were 2, 5, and 7, respectively. The field width, pitch, and modulation factor (MF) used in tomotherapy were 2.5 cm, 0.32, and 3.5, respectively. A fraction size of 2 Gy was chosen as daily dose. For the radiation dose to the normal tissues, an isodose line of 0.5 Gy was designed to represent the off-target, general low-dose area during daily treatment. Notably, there is no dose-painting result in 2DRT planning, the technique in the past era. Patient data are collected with the approval of the Institutional Review Board of Far Eastern Memorial Hospital (FEMH-IRB-100163-F).

### Materials and reagents

The 5-FU/CDDP and high-performance liquid chromatography (HPLC)-grade methanol were purchased from Sigma Chemicals (St. Louis, MO, USA) and Tedia Company, Inc. (Fairfield, OH, USA), respectively. Milli-Q grade (Millipore, Bedford, MA, USA) water was used for the preparation of solutions and mobile phases.

### Animals and sample preparation

**Figure 1:**
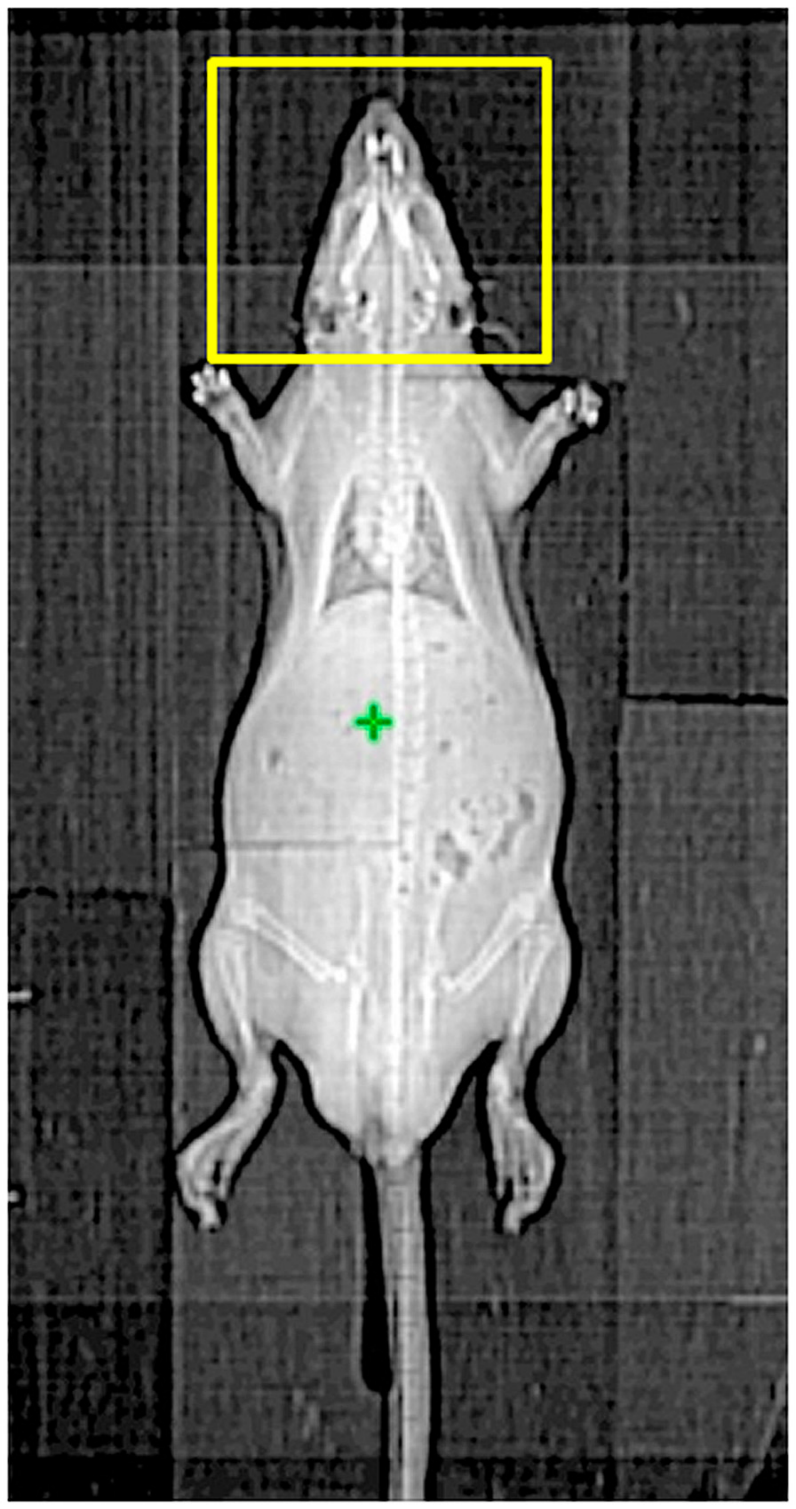
**Computed tomography was used for simulation of the head and neck field.** The cranial margin was set at 5 mm above the head and the caudal margin was set above the head of humerus. Conventional radiotherapy was used to deliver the radiation dose via the anterior-posterior (AP) and PA portals.

Adult, male Sprague–Dawley rats (300 ± 20 g body weight) were provided by the Laboratory Animal Center at National Yang-Ming University (Taipei, Taiwan). The rats were anesthetized with urethane 1 g/ml and α-chloralose 0.1 g/ml (1 ml/ kg, i.p.), and were immobilized on a board to undergo computed tomography for simulation of the one phase of head and neck RT field. The cranial margin was set at 5 mm above the head and the caudal margin was set above the head of humerus. (Figure 1) The conventional technique with anterior-posterior (AP) and PA portal by 6-MV X-ray was delivered by an linear accelerator (Varian Medical Systems, Palo Alto, CA). The experimental animals were randomized to three separate experiments for no drug, CDDP and 5-FU administration and each experiment included at least 6 rats for each with control (0 Gy), 0.5, and 2 Gy group. All experimental animal surgery procedures were reviewed and approved by the animal ethics committee of Far Eastern Memorial Hospital, Taiwan (FEMH- 100-1-18-A).

Jarugula *et al*. [29] proved that the dose-normalized area under the curve (AUC) is significantly higher after administration of 100 mg/kg than after 50 mg/kg or 10 mg/kg. Thus, we chose 100 mg/kg as a feasible 5-FU dose in rats for examination of 5-FU pharmacokinetic parameters, based on previous reports [17,29].

The LD50 of CDDP for rat with oral, intraperitoneal, subcutaneous, intravenous is 25, 7, 8 and 11 mg/kg, respectively [30]. Therefore, we chose 50% of LD50 of intravenous CDDP, 5 mg/kg, as a feasible dose in rats for examination of CDDP pharmacokinetic parameters.

Twenty hours after RT, the rats were administered 5-FU at 100 mg/kg or 5 mg/kg CDDP in 2 mL of normal saline by intravenous infusion into the femoral vein over a 2-min period. A 150-μL blood sample was withdrawn from the jugular vein with a fraction collector according to a programmed schedule at 5, 15, 30, 45, 60 min, 1.5, 2, 2.5, and 3 h following drug administration. After centrifugation and vortexing, the upper organic layer was transferred to a new tube and evaporated to dryness under flowing nitrogen. The dried residue was reconstituted with 50 μl of Milli-Q water (Millipore). A 20-μL aliquot of the solution was injected to the HPLC-UV system.

### Liquid chromatography

#### 5-FU

Chromatographic analysis is performed on a Model LC-20AT HPLC system (Shimadzu, Tokyo, Japan) equipped with a Model SPD-20A wavelength UV detector, SIL-20 AC autosampler, and an LC Solution data processing system. A LiChroCART RP-18e column (Purospher, 250 mm, 5 μm, Merck, Darmstadt, Germany) with a LiChroCART 4–4 guard column is used for separation. The mobile phase comprised 10 μM potassium phosphate-methanol (99: 1, v/v, pH 4.5 adjusted by 85% phosphoric acid), and the flow rate of the mobile phase is 1 ml/min. The detection wavelength is set at 266 nm. Under these conditions, the retention time of 5-FU is 5.4 min. The linearity of calibration curves is demonstrated by the good determination coefficients (r2) obtained for the regression line. Good linearity is achieved over the range of 0.01–5 μg/ml, with all coefficients of correlation greater than 0.998. All samples are freshly prepared, including the standard solutions, from the same stock solution (5 mg/mL). The 0.01-μg/mL limit of quantification is defined the lowest concentration on the calibration curve that could be measured routinely with acceptable bias and relative SD. The overall mean precision, defined by the relative SD, ranged from 0.2% to 11.0%. Analytical accuracy is expressed as the percentage difference of the mean observed values compared to known concentrations varying from -10.0% to 14.0%. The recovery results for concentrations of 0.1-10 μg/mL are 92.0%-94.0%.

#### CDDP

The HPLC system consist of a chromatographic pump (LC-20AT, Shimadzu, Kyoto, Japan), autosampler (SIL-20AT, Shimadzu), diode array detector (SPD-M20A, Shimadzu), and degasser (DG-240). A reversed-phase C18 column (4.6 × 250 mm, particle size 5 μm, Eclipse XDB, Agilent, Palo Alto, CA, USA) is used for the HPLC separation. The mobile phase is composed of acetonitrile-10 mM monosodium phosphate (pH 3.0 adjusted by orthophosphoric acid) (70:30, v/v) at a flow-rate of 1.0 ml/min. The chromatographic run time is 13 min and the detection wavelength is set at 254 nm. The mobile phase is filtered through a 0.45 μm Millipore membrane filter and degassed by sonication 2510R-DTH (Bransonic, CT, USA) before use. The stock solution of CDDP in 50% acetonitrile (500 μg/ml) is diluted with 50% acetonitrile to make serial concentrations of the working standard solutions (1, 5, 10, 50 and 100 μg/ml). Plasma is separated by centrifuging the blood sample at 6000 rpm for 10 min at 4 OC. Calibration standards are prepared by 5 μl of the working standard solution spik with 45 μl of blank plasma and bile, and then added 10 μl of freshly prepared 10% diethyldithiocarbamate in 0.2 N sodium hydroxide into each sample. These samples are put into the water bath at 45°C for 30 min to form the derivatization of CDDP. The 100 μl of internal standard solution (containing 10 μg/ml of magnolol dissolved in acetonitrile) is added to the derivative samples for protein precipitation. The samples are vortexed and centrifuged at 12,000 rpm for 15 min, after which, 20 μl of supernatants are collected and analysed by the HPLC system. The calibration curves are represented by the peak areas ratio of the CDDP to internal standard spiked in blank samples vs. the concentration of CDDP. The limits of detection (LOD) and the limit of quantification (LOQ) are defined as a signal-to-noise ratio of 3 and the lowest concentration of the linear regression, respectively. The 0.01-μg/mL limit of quantification is defined the lowest concentration on the calibration curve that could be measured routinely with acceptable bias and relative SD. The overall mean precision, defined by the relative SD, ranged from 0.6% to 2.2%. Analytical accuracy is expressed as the percentage difference of the mean observed values compared to known concentrations varying from -7.4% to 1.0%. The recovery results for concentrations of 0.1-10 μg/mL are 80.7%-82.9.0%.

### Pharmacokinetics and data analysis

Pharmacokinetic parameters such as the AUC for concentration *vs.* time, terminal elimination phase half-life (t_1/2_), maximum observed plasma concentration (Cmax), mean residence time (MRT), total plasma clearance (CL), volume of distribution at steady state (Vss), and the elimination constant (Kel) were calculated by the pharmacokinetics calculation software WinNonlin Standard Edition, Version 1.1 (Scientific Consulting, Apex, NC, USA) using a compartmental method.

### Statistical methods

The results are presented as means ± standard deviations. Differences in actuarial outcomes between the groups were calculated using one-way analysis of variance (ANOVA), with post hoc multiple comparisons. All analyses were performed using the Statistical Package for the Social Sciences, version 12.0 (SPSS, Chicago, IL, USA).

## Results

### Comparison of treatment plans for different radiation dosing techniques

**Figure 2:**
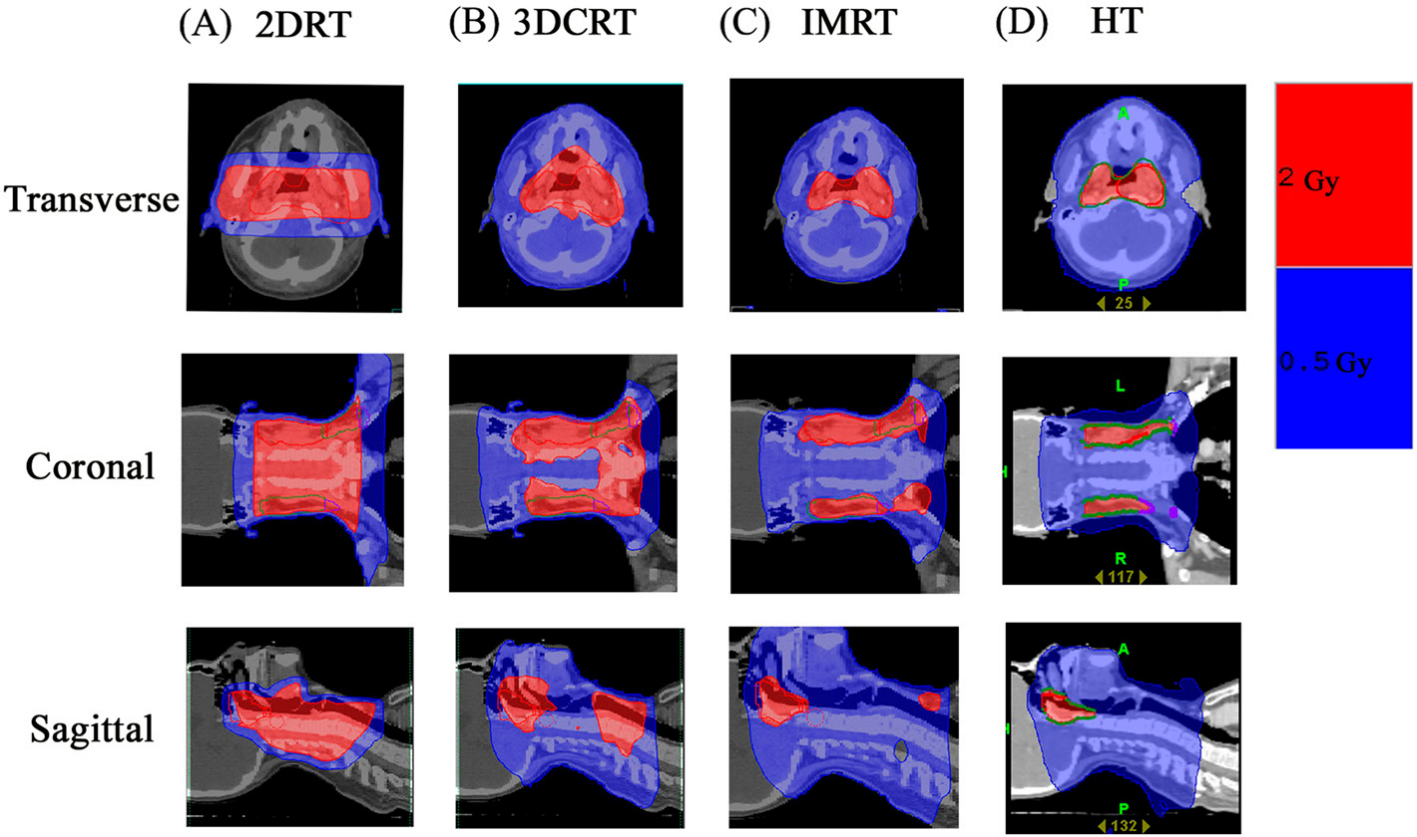
**An example of isodose distribution using different irradiation techniques delivering 2 Gy to the tumor bed for one nasopharyngeal carcinoma patient with transverse, coronal and sagittal view. A)** The conventional radiation therapy (2DRT). **B)** Three-dimensional conformal radiotherapy (3DCRT). **C)** Intensive modulated radiotherapy (IMRT). **D)** Helical tomotherapy (HT).

A representative example of isodose distribution with 2 Gy to the targets using different techniques is illustrated in Figure 2. It suggests that low dose area generously deposited around target volume especially in the era of advanced, conformal radiation techniques.

### Plasma and bile pharmacokinetic parameters of 5-FU and CDDP with head and neck irradiation

#### 5-FU

**Figure 3:**
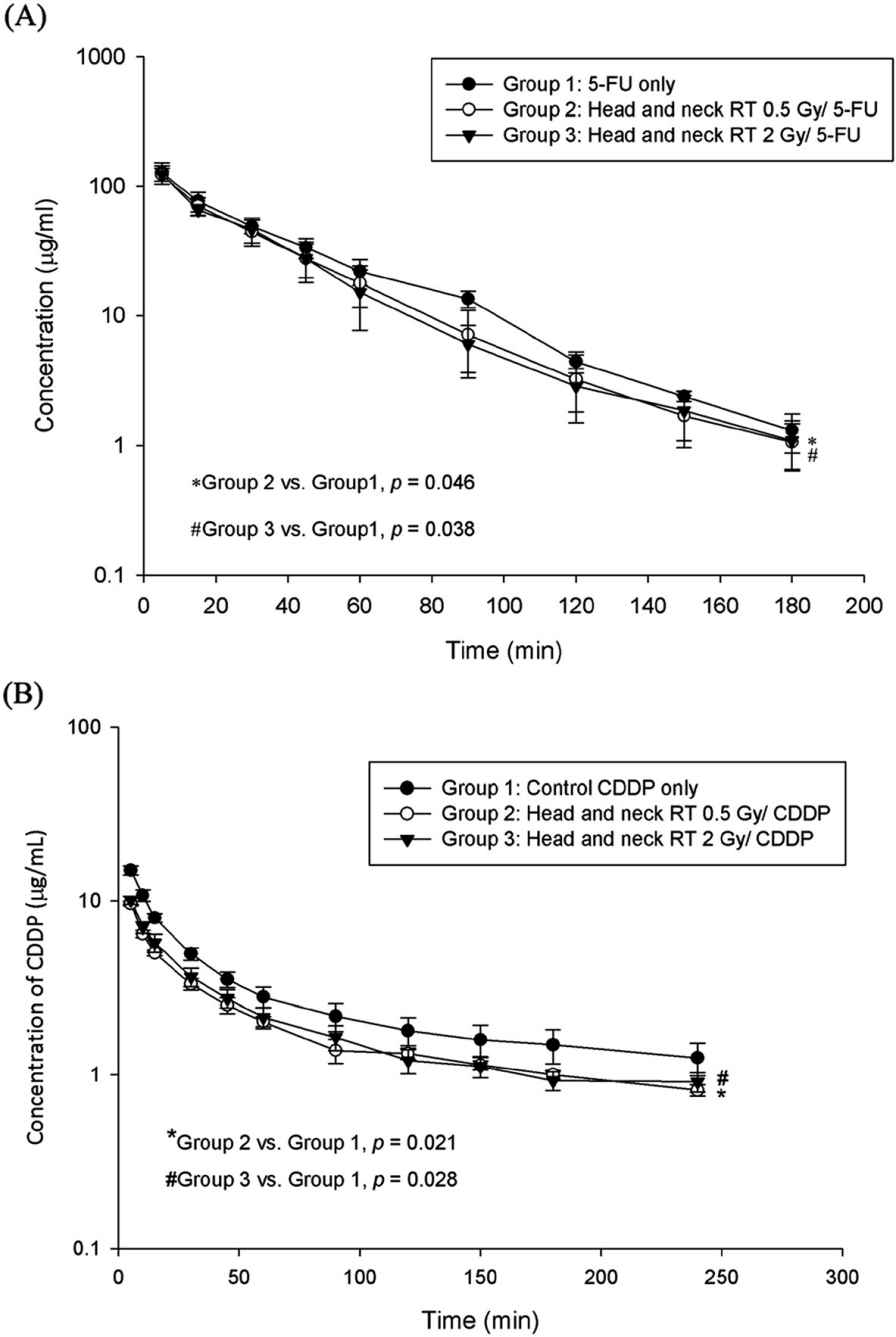
**The area under the plasma concentration *vs. *time curve (AUC) of (A) 5-FU 100 mg/kg (B) Cisplatin 5 mg/kg to rats in the control, 0.5 and 2 Gy groups with head and neck irradiation.**

To verify that local RT modulated the systemic pharmacokinetics of 5-FU, we established an experimental model using CT-based planning and one phase of head and neck irradiation in rats merged to our pharmacokinetics assay system. (Figure 1) Of special interest, radiation at 2 Gy simulating daily treatment dose to compare with low dose radiation at 0.5 Gy representing a dose deposited in the generous, off-target area in clinical practice. Intriguingly, we found that irradiation markedly reduced the AUC of 5-FU in rats by 16.9% at 0.5 Gy (*p* = 0.046) and 15.9% at 2 Gy (*p* = 0.038), respectively. (Figure 3A) Irradiation significantly reduced MRT, and by contrast, increased the CL of 5-FU when compared to non-irradiated controls (Table 1). There was no significant difference in the values of Cmax, Vss and t_1/2_ within any group.

**Table 1:** Pharmacokinetic parameters of 5-Fluorouracil (100 mg/kg, i.v.) in rats plasma after head and neck irradiation with and without 0.5 and 2 Gy

**Parameters**	**Controls**	**One phase of head and neck irradiation**	
	**0 Gy**	**0.5 Gy**	**2 Gy**
AUC (min μg/mL)	4748 ± 360	4017 ± 358*	3993 ± 191*
t_1/2_ (min)	33.1 ± 11.5	35.5 ± 9.7	39.0 ± 5.6
Cmax (μg/mL)	165 ± 35	165 ± 17	167 ± 39
MRT (min)	37.0 ± 2.8	30 ± 4.5*	29 ± 2.9*
CL (mL/kg/min)	21.2 ± 1.7	25 ± 4.8	27 ± 5.7*
Vss (mL/kg)	785 ± 96	784 ± 88	815 ± 130

*AUC* area under the plasma concentration, *vs.* time curve, *t*_*1/2*_ terminal elimination phase half-life, *Cmax* maximum observed plasma concentration, *MRT* mean residence time, *CL* total plasma clearance, *Vss* volume of distribution at steady state.

*The mean difference is significant at the 0.05 level in comparison to the control group.

**Figure 4:**
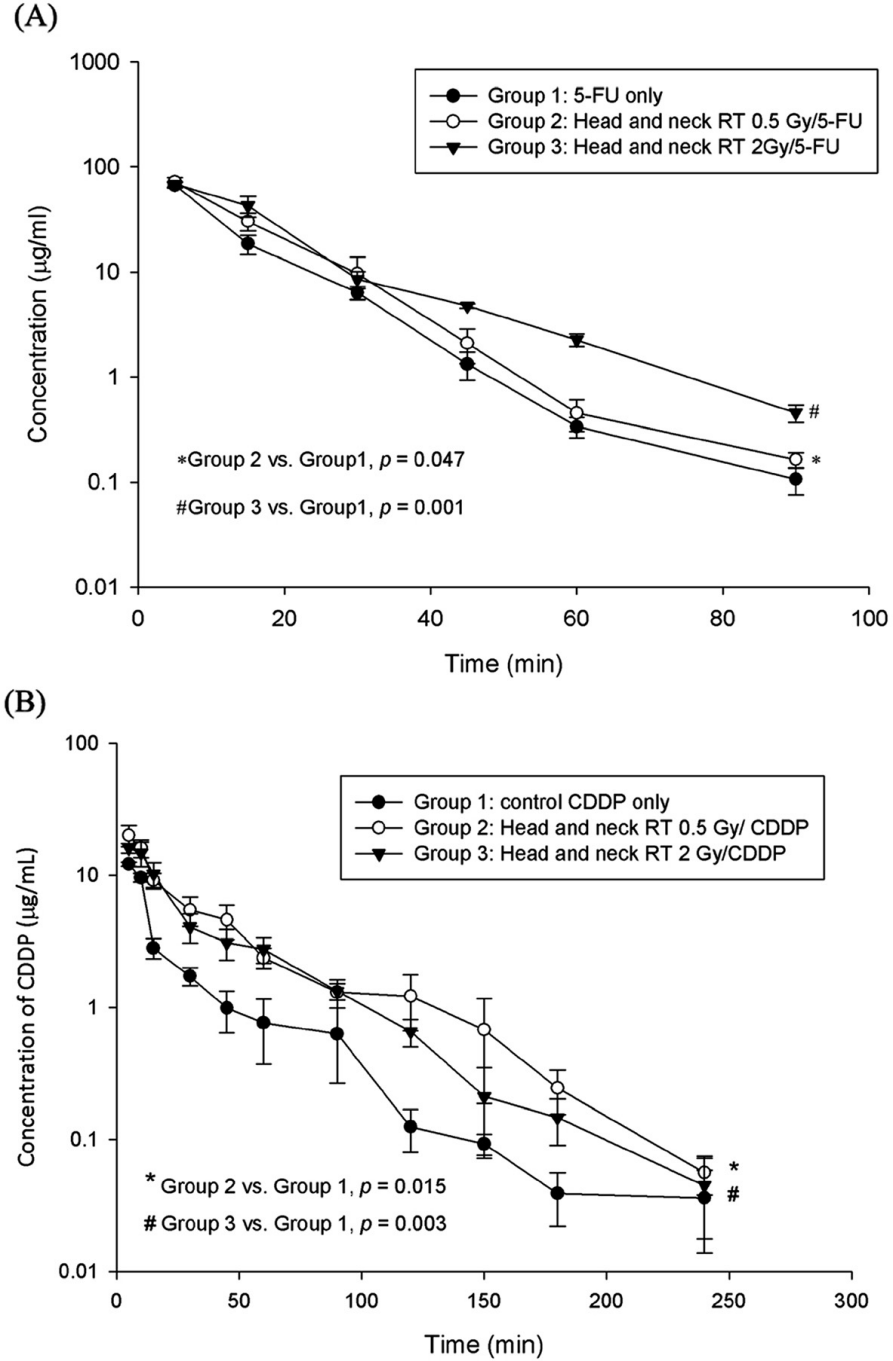
**The area under the bile concentration *vs. *time curve (AUC) of (A) 5-FU 100 mg/kg (B) Cisplatin 5 mg/kg to rats in the control, 0.5 and 2 Gy groups with head and neck irradiation.**

The AUC of 5-FU in bile of rats after head and neck irradiation markedly increased by 12.2% at 0.5 Gy (*p* = 0.047) and 25.0% at 2 Gy (*p* = 0.001). (Figure 4A) Head and neck irradiation significantly decreased Cmax and CL, and in contrast, increased MRT of 5-FU, when compared to non-irradiated controls. Of interest, 2-Gy irradiation decreased Cmax (*P* = 0.004) and CL (*P* = 0.001), and in contrast, increased MRT (*P* < 0.001) of 5-FU to an extent greater than that of the 0.5-Gy group. There was no statistically significant difference between the 0.5-Gy and control groups for T_1/2_, clearance value, MRT and Vss. (Table 2).

**Table 2:** Pharmacokinetic parameters of 5-Fluorouracil (100 mg/kg, i.v.) in rats bile after head and neck irradiation with and without 0.5 and 2 Gy

**Parameters**	**Controls**	**One phase of head and neck irradiation**	
	**0 Gy**	**0.5 Gy**	**2 Gy**
AUC (min μg/mL)	1218 ± 22	1366 ± 86^*^	1522 ± 111^*^
t_1/2_ (min)	9.4 ± 0.5	9.2 ± 0.2	13.3 ± 1.1^*^
Cmax (μg/mL)	129 ± 22	104 ± 14^*^	88 ± 17^*^
MRT (min)	10.0 ± 1.0	10.7 ± 0.5	14.5 ± 0.6^*^
CL (mL/kg/min)	80.3 ± 2.3	76.3 ± 5.4	65.6 ± 4.5^*^
Vss (mL/kg)	812 ± 96	820 ± 66	952 ± 69

*AUC* area under the plasma concentration, *vs.* time curve, *t*_*1/2*_ terminal elimination phase half-life, *Cmax* maximum observed plasma concentration, *MRT* mean residence time, *CL* total plasma clearance, *Vss* volume of distribution at steady state.

*The mean difference is significant at the 0.05 level in comparison to the control group.

#### CDDP

Head and neck irradiation also modulated the AUC of CDDP in rats by decreasing 33% at 0.5 Gy (*p* = 0.021) and 29% at 2 Gy (*p* = 0.028), respectively. (Figure 3B) By contrast, increased the CL of CDDP at 0.5 Gy (*p* = 0.02) and 2 Gy (*p* = 0.019) when compared to non-irradiated controls (Table 3). There was no significant difference in the values of MRT, Vss and t_1/2_ within any group.

**Table 3:** Pharmacokinetic parameters of CDDP (5 mg/kg, i.v.) in rats plasma after with or without head & neck irradiation at control, 0.5 and 2 Gy

**Parameters**	**Controls**	**One phase of head and neck irradiation**	
	**0 Gy**	**0.5 Gy**	**2 Gy**
AUC (min μg/mL)	722 ± 164	484 ± 52*	511 ± 138*
t_1/2_ (min)	269 ± 108	215 ± 88.5	172 ± 44.6
Cmax (μg/mL)	15.0 ± 2.2	9.6 ± 0.7*	10.1 ± 1.3*
MRT (min)	291 ± 131	248 ± 97.5	249 ± 116
CL (mL/kg/min)	4 ± 1.2	6.8 ± 1.1*	6 ± 0.9*
Vss (mL/kg)	1233 ± 470	1621 ± 520	1626 ± 683

*AUC* area under the plasma concentration, *vs.* time curve, *t*_*1/2*_ terminal elimination phase half-life, *Cmax* maximum observed plasma concentration, *MRT* mean residence time, *CL* total plasma clearance, *Vss* volume of distribution at steady state.

*The mean difference is significant at the 0.05 level in comparison to the control group.

Head and neck irradiation markedly increased the AUC of CDDP in bile of rats by 0.5 Gy (*p* = 0.015) and at 2 Gy (*p* = 0.003), respectively (Figure 4B). When compared to non-irradiated controls, head and neck irradiation significantly increased CL of CDDP at 0.5 Gy (*p* = 0.001) and 2 Gy (*p* = <0.001), respectively. There was no statistically significant difference for Cmax, T_1/2_, MRT and Vss. (Table 4).

**Table 4:** Pharmacokinetic parameters of CDDP (5 mg/kg, i.v.) in rats bile after head and neck irradiation with and without 0.5 and 2 Gy

**Parameters**	**Controls**	**One phase of head and neck irradiation**	
	**0 Gy**	**0.5 Gy**	**2 Gy**
AUC (min μg/mL)	262 ± 52	542 ± 192*	611 ± 66*
t_1/2_ (min)	32 ± 15	27 ± 5.1	28 ± 7.8
Cmax (μg/mL)	12 ± 1.0	19 ± 10	18 ± 2.8
MRT (min)	28 ± 9.4	33 ± 8.3	32 ± 11.6
CL (mL/kg/min)	19 ± 3.4	10 ± 2.9*	8.2 ± 0.9*
Vss (mL/kg)	544 ± 202	337 ± 150	257 ± 94

*AUC* area under the plasma concentration, *vs.* time curve, *t*_*1/2*_ terminal elimination phase half-life, *Cmax* maximum observed plasma concentration *MRT* mean residence time, *CL* total plasma clearance, *Vss* volume of distribution at steady state.

*The mean difference is significant at the 0.05 level in comparison to the control group.

## Discussion

From 2DRT to arc therapy techniques, these therapies are supposed to produce greater target dose conformity and better critical organ sparing effects, allowing target dose escalation, with lower toxicity to normal tissues [10,31-33]. However, the more beam arranged the more accompanied low-dose distribution to the torso are noted. Figures 1 and 2 illustrated the example of isodose distribution with 2 Gy to the targets in head and neck patient using the different techniques. We noted that more than 50% of the normal organ was exposed to 0.5 Gy during daily 2-Gy radiation treatments, except when using 2DRT to treat patients. These dose-painting characteristics of modern radiation technique are also noted in other part of body’s treatment [17]. It suggests that the low-dose radiation area generously deposits around the target volume, especially when advanced, conformal radiation techniques are used.

In the previous studies, we confirm that abdominal or pelvic irradiation modulates the systemic PK of 5-FU at 0.5 Gy, off-target area in clinical practice, and at 2 Gy, the daily treatment dose for target treatment in an experimental rat model [17,18]. Additionally, the RT-PK phenomena are related to the releasing of MMP-8 [18]. The liver and kidneys are excluded in the previous study to rule out the influences caused by irradiated these parts because of 80% of 5-FU is catabolized by the liver [34,35] and 10% to 20% of 5-FU is excreted by the kidneys [36]. Furthermore, polymorphonuclear neutrophils (PMNs) are the main source of MMP-8 in humans and mice [37,38] and 5-FU often induces leukocyte infiltration of rat small intestine by the gastrointestinal toxicity [19]. Additionally, 5-FU is highly absorbable in a gastric emptying-limited manner with first-pass metabolism concerning [39] and the uptake of 5-FU in intestinal also influences by passive transport and carrier-mediated transport [20]. Therefore, it is difficult to specify the PK of 5-FU modulated by RT is a general phenomena especially when RT field still cover partial intestines.

In the current study, only HN was irradiated for PK analysis. (Figure 1) the AUC of 5-FU in rats was reduced not only at 0.5 Gy but also at 2 Gy. (Figure 3A) Additionally, the AUC of 5-FU in bile of rats after HN irradiation increased for both irradiation groups. (Figure 4A) Furthermore, the CL rate in irradiation groups were increased in plasma and decreased in bile study. (Tables 1 and 2) It suggests that local irradiation may modulate the systemic PK of 5-FU and facilitate the excretion of 5-FU discernible to the area irradiated.

CDDP and 5-FU combination treatments are reported to be one of the most active chemotherapeutic regimens for the patients with head and neck squamous cell carcinoma [4-6]. Although both CDDP and 5-FU induce apoptosis through caspase activation, the apoptosis induced by CDDP was reported to be involved in caspase-9 [40], in contrast to the involvement of caspase-1, -3 and -8 in 5-FU-induced apoptosis [41]. Additionally, CDDP is known to intercalate into DNA at any phase of the cell cycle [42,43], causing the cytotoxic effect and reducing the cellular viability [44]. 5-FU inhibits the function of RNA or DNA synthesis [45,46] and causes the cytostatic effect accompanied by the growth arrest at the G1/S boundary of the cell cycle [47]. In this study, the AUC of CDDP in plasma was decreased no matter at 0.5 Gy or 2 Gy (Figure 3B) and increased the CL of CDDP when compared to non-irradiated controls (Table 3). Additionally, the AUC of CDDP in bile of rats was increased by 0.5 Gy and 2 Gy. (Figure 4B) (Table 4). It suggests that similar modulation also between head and neck irradiation and the systemic PK of CDDP with facilitating the excretion of CDDP. Irradiation is not only modulating PK of 5-FU but also modulating PK of CDDP. Because this RT-PK phenomenon exists in drugs with different mechanisms of action, it implicates that RT may modulate PK in a way independent to downstream drug targets.

There are some limitations in the current study. First, the role of MMP-8 as observed in abdominal and pelvic RT is not proved in the current study. The role of abdominal and pelvic RT-induced MMP-8 in modulating 5-FU pharmacokinetics has been demonstrated. Whether this soluble factor has an impact on head and neck RT-modulated pharmacokinetics of 5-FU and CDDP remains to be determined. Second, this study is the effects of irradiation followed by chemotherapy with one-shot design rather than fractionation RT as daily practice. For proof of concept, we designed the use of one shot irradiation followed by chemotherapy. In clinical use of fractionated RT and periodically concurrent chemotherapy, the previous daily fraction of RT may modulate the PK of chemotherapeutics administered in the consecutive day. In the conducting clinical trial, we planned to validate this RT-modulated PK phenomenon in patients receiving CCRT with fractionated RT. Third, this study is the effects of irradiation followed by chemotherapy but concurrent chemotherapy with radiotherapy is the usual way that applied in the clinical practice. However, patients with rectal cancer who receive preoperative radiotherapy, adding fluorouracil-based chemotherapy preoperatively or postoperatively has no significant effect on survival. Chemotherapy, regardless of time sequence, confers a significant benefit with respect to local control [48]. Additionally, fluorouracil and cisplatin to radiotherapy within 16 hours after the first radiation fraction was administered that significantly improved the survival rate of women with locally advanced cervical cancer [49]. These data explain the importance of adding chemotherapy to RT but rather than time sequence of drug delivering. The findings for modulation of drug PK by local RT, as demonstrated in previous and this study, may provide a clue and a research platform to clarify this controversy. Finally, the data of pharmacokinetics for head and neck cancer patients during CCRT is not collected in the current study. Thus, the further study for RT-PK phenomena of 5-FU and CDDP in head and neck cancer patients is warranted in the future.

## Conclusions

To our best knowledge, this is the first study to prove that head and neck irradiation without liver, kidneys and intestines irradiation that can significantly modulate the systemic pharmacokinetics of 5-FU and CDDP at dosage levels for both the target (2 Gy) and off-target areas (0.5 Gy). This study may provide an experimental clue to understand the unclear biological effects of generous, low-dose RT in the era of highly conformal RT in head and neck treatment. For head and neck irradiation with concurrent 5-FU and CDDP, this unexpected RT-PK phenomena may provide a reference for adjustment of drug administration during CCRT and is worthy of further investigation in clinical practice.

## Competing interest

The authors declare that they have no competing interests.

## Authors’ contributions

CH Hsieh participated in the design of the study, performed the radiation and pharmacokinetic experiments, and wrote the manuscript. ML Hou and MH Chiang helped CH Hsieh to do some experiments. HC Tai and HJ Tien was responsible for the radiation planning. LY Wang helped to design the experiments. TH Tsai and YJ Chen initiated, organized and supervised all the work, including the manuscript. All authors read and approved the final version of this manuscript.
